# Spatial Analysis of Citizens’ Environmental Complaints in China: Implications in Environmental Monitoring and Governance

**DOI:** 10.3390/ijerph18189674

**Published:** 2021-09-14

**Authors:** Xuepeng Ji, Daoqin Tong, Lisha Cheng, Xiaowei Chuai, Xiyan Mao, Binglin Liu, Xianjin Huang

**Affiliations:** 1 School of Geographic and Oceanographic Sciences, Nanjing University, Nanjing 210023, China; dg1727011@smail.nju.edu.cn (X.J.); mg1927027@smail.nju.edu.cn (X.C.); mxy@nju.edu.cn (X.M.); DG1827018@smail.nju.edu.cn (B.L.); 2 School of Geographical Sciences & Urban Planning, Arizona State University, Tempe, AZ 85287, USA; Daoqin.Tong@asu.edu; 3 College of Geography and Environment, Henan University, Kaifeng 475004, China; chengls487@nenu.edu.cn

**Keywords:** citizen environmental complaints, hotline and internet, spatial characteristics, influencing factors, spatial regression models, China

## Abstract

Citizen environmental complaints play a key role in China’s current environmental monitoring network and environmental governance system. Based on 5796 cases of environmental complaints lodged by citizens via hotline and the internet to the MEP of China, we examined the spatial characteristics and influencing factors of citizen complaints for the period of 2013–2017 using spatial analysis methods and spatial econometric models. The roles of citizen complaints in the two systems were then reevaluated. The results show that, among all cases, 75.88% of cases were identified as verified complaints, while nearly a 25% noisy rate directed large amounts of inspection resources to be utilized in response to nonverified cases. Air pollution received the most attention by citizens in China, accounting for 67.22% of total cases. The hotspots of citizen complaints were mostly distributed in the three major national urban agglomerations in China. We found that industrial wastewater and SO_2_ were positively associated with the likelihood of citizens filing complaints, while the effect of industrial soot/dust emission was insignificant. Citizen complaints might be triggered by certain, but not all, forms of pollutants, even though highly visible particulate pollutants did not necessarily induce corresponding complaints. Moreover, the negative relationship between citizen complaints and per capita GDP revealed the unbalanced geographical pattern between economical development and environmental quality. The proliferation of the internet greatly facilitated citizens lodging complaints through various ways. The synergy mechanism between citizen environmental complaints and other parts in China’s environmental monitoring and governance system should be established in the future.

## 1. Introduction

Since the Chinese reform and opening up in 1978, China has experienced an unprecedented industrialization and urbanization process over the past forty years. While such an important historical process has resulted in high-speed economic growth and continuously improved life quality, severe environmental pollution and ecological degradation problems have occurred across the country and are frequently reported through multiple media channels. According to the Report on the State of the Ecology and Environment in China in 2017, 239 cities failed to meet national air quality standards, accounting for 70.7% of all cities [1]. Furthermore, 66.6% of 5100 groundwater monitoring sites exhibited poor or very poor water quality; the total area of counties with relatively poor or poor ecological environmental quality took up 33.5% of total land area [1]. This, in turn, could endanger human health and safety, and further challenge the sustainable development of the economy and society in China.

In order to effectively address these issues of environment and ecology, a multi-agent system for environmental governance has been gradually established in China over the years, mainly including governments, enterprises, social organizations and public participation [2]. Under this mechanism, public participation plays an important bridging role, linking other parts of the system through empowering civil rights and encouraging citizens to participate in environmental protection [3,4,5]. Correspondingly, the government of China issued a series of laws and regulations to ensure their citizens’ rights to participate in environmental governance [6]; for example, the Environmental Protection Law was issued in 1989 and amended in 2014. Other pertinent laws and regulations include the Decision of the State Council on Several Issues Concerning Environmental Protection (1996), Environmental Impact Assessment Law (2003), Measures for Public Participation in Environmental Protection (2015), and Guiding Opinions on Building a Modern Environmental Governance System (2020). Furthermore, National Five-Year Plans and several action plans for the prevention and control of major forms of pollution (i.e., air, water, soil pollution) also emphasized and elaborated the function and importance of public participation for environmental protection and management.

Of all the modes of public participation, citizen environmental complaints are undoubtedly the most popular and widely-used mode for individuals or communities protecting their environmental interests and public safety [7]. Other modes, for instance, environmental protests, internet public opinions, public hearings in environmental impact assessments, environmental non-government organizations (ENGOs), NPC motions and CPPCC proposals, together with citizen complaints jointly constitute the main body of the public participation subsystem in the environmental governance system [8,9]. The Chinese state has set up its own environmental complaint system since the early 1990s, namely the ‘Huanjing Xinfang’ system, a kind of system which literally means citizens can lodge their complaints regarding environmental issues by means of writing letters or visiting environmental agencies directly [10,11]. It should be noted that traditional channels for citizens lodging complaints (e.g., writing letters or visiting the authorities in early days) has been gradually replaced by emerging ways (e.g., dialing a hotline, reporting online or, more recently, simply via smartphone apps) in the past three decades. In reality, the availability of multiple and convenient channels also led to a surge in the number of complaints and people involved. This change makes necessary the reevaluation of the role of citizen environmental complaints in China’s current environmental governance system.

Although the efficacy of citizen environmental complaints on pollution abatement is still contentious, plenty of studies show that it is conducive to environmental governance. This can be understood by assessing the role that citizen complaints play in two closely connected but distinct circumstances. In the first place, a number of studies examined the effectiveness of citizen complaints on environmental performance, regarding citizen complaints as a policy tool for public participation in the environmental governance system. Some studies suggested that environmental complaints in China had positive influences on pollution control [2,5,12], while other scholars held the opposite position, suggesting that it had limited or even no significant effects on improving regional environmental quality [5,13,14]. It is worth nothing that the usefulness of citizen complaints may vary widely in terms of pollutant types and complaint methods at the aggregate level. Moreover, the complaints may also exert various influences on environmental laws, regulations or enforcement of the government, thereby indirectly prompting the enterprise behaviors to comply with local environmental policies and improve the overall environment.

In the second place, as a special monitoring instrument, citizen environmental complaints can also provide useful but noisy information in reflecting environmental quality and health. Over the past few decades, China has progressively established a relatively well-developed monitoring network to report eco-environmental dynamics. In spite of this, the network is far from monitoring all sorts of pollution sources, especially for those small-medium enterprises dispersedly distributed in remote or rural areas in China [10]. Therefore, regulators may solicit complaints from the public damaged by pollution so as to allocate inspection resources, because local residents are often more familiar with neighboring polluters located in their living surroundings [15]. This kind of cost-effective approach for direct monitoring, however, often contains noisy information that requires further investigation and verification on the spot [16,17]. In general, the public has a propensity to complain about those polluting issues with visible and odorous pollutants [15]. In addition, other possible factors, such as forms of pollutants, regional disparity and citizens’ environmental awareness, may also contribute to the cognitive bias of environmental information in indicating actual pollution problems or polluted regions. 

Nevertheless, previous research on the role of citizen environmental complaints and the factors mobilizing citizens to file complaints on environmental issues is still limited. Theoretically, if there is no environmental pollution, there are no environmental complaints. Thus, a handful of studies suggested that citizen complaints were significantly associated with pollution discharges in China using provincial level statistical datasets [10,15,18,19]. Complaints might be affected by certain, but not all, forms of harmful pollutants, such as high-visible dust particulates in the air [15], industrial waste water [18,19], chemical oxygen demand and SO_2_ emissions [10]. In addition, those socio-economic factors that may drive people’s awareness and behaviors to lodge complaints were also examined and discussed in depth. Dasgupta and Wheeler investigated and estimated the relationship between environmental complaints and socio-economic factors in China using a provincial panel data set over the period 1991–1993 [15]. They found that higher income and education levels increased the incidence of citizens filing complaints, which may lead to disproportionate allocation of inspection resources to those who live in areas with better economic and educational conditions. Based on econometric models and statistical data at the provincial level in China, some studies have suggested that complaints were significantly related with household income or per capita GDP [10,19], education level and population size [20]. Furthermore, several studies were performed to assess the influencing factors of environmental complaints in relation to livestock operations or agricultural spills in North America [16,17,21]. For example, Weersink and Raymond examined the influence of local environmental regulations and other factors on the possibility of both farm spills and complaints in southwestern Ontario, Canada for the period 1993–1996 [16]. Their results suggested that agricultural spills, education and income had a significantly positive impact on the number of complaints and indicated that the complaints could be used by the local regulators to identify problem regions, though the information signals would be noisy. Additionally, this kind of environmental behavior may differ considerably from person to person in terms of environmental psychology. Zhang et al. explored the social psychological antecedents of citizen environmental complaints by using the norm activation model and questionnaire survey data in China [7,11], which could aid in the understanding of what microscopic factors drove citizens to file complaints to environmental agencies.

To our best knowledge, however, most of the existing literature studying citizen environmental complaints in China merely utilized traditional methods of complaints from statistical data as a proxy index (e.g., the number of visits or letters) at macro levels (e.g., national or provincial levels) [9,10,12,15,19,20]. Additionally, the typical characteristics of environmental complaints, including its accuracy, types of complaints, and noisy problems, were rarely analyzed or discussed in previous literatures. The purpose of this paper is to reevaluate the role of citizen environmental complaints using cases of citizen environmental complaints via hotline and the internet from the MEP of China for the period 2013–2017. In order to do so, we first describe the typical characteristics of the accuracy and types of environmental complaints. The spatial patterns and influencing factors are then identified and presented at prefecture-level city scales by employing spatial analysis methods and spatial econometric models, and finally the role of the complaints is assessed in China’s current environmental governance system and environmental monitoring network.

The rest of the paper is organized as follows: Section 2 describes data sources and research methods selected. Section 3 presents the results of statistical characteristics, spatial patterns and spatial regression models of citizen environmental complaints in China. The potential influencing factors of citizen complaints regarding environmental problems together with its roles in China’s current environmental governance and monitoring systems are then discussed in Section 4. Finally, we summarize major findings and policy implications in Section 5.

## 2. Materials and Methods

### 2.1. Data Sources and Processing

In this study, the data of citizen environmental complaints were obtained for the period 2013–2017, collected from the 12369 Network Reporting Platform [22]. This reporting platform was established to facilitate citizens lodging complaints via hotline and the internet regarding environmental pollution or ecological destruction issues by the Ministry of Environmental Protection of China (MEP, known as the Ministry of Ecology and Environment of China since 2018) in 2011. This dataset is based on individual cases that provided detailed information for each case, including serial number, year, month, province, involved enterprises, existing environmental problems, and disposal information. It should be noted that the method of data collection was citizens across the country lodging their complaints directly to the MEP instead of local environmental agencies, and Hong Kong, Macao, and Taiwan were not included in this data set due to data availability. After collection, the geographic location of all cases was acquired by geocoding technique based on the name and address of involved enterprises. Then, different types of environmental pollution for all cases were grouped into 6 categories (i.e., air pollution, water contamination, solid waste pollution, noise pollution, illegal production, and others) for further analysis. According to the attributes of existing environmental problems and disposal information, we further identified whether these cases had been verified or not, or to say, which cases had been proven to be valid or not by the investigation of local agencies or regulators.

To understand the potential factors behind environmental complaints filed by citizens in China, we selected 10 independent variables with respect to socio-economic status, environmental pollution, and environmental awareness. The selected variables were all at the prefecture level and were mainly obtained from the China City Statistical Yearbook, and the missing values were completed through the Provincial Statistical Yearbook and the Statistical Bulletin on National Economic and Social Development in China.

### 2.2. Methods

#### 2.2.1. Spatial Autocorrelation and Hot Spot Analysis

Identifying geographic patterns is of great significance to better understand how geographic phenomena behave across space and over time [23]. Spatial autocorrelation is a major feature of geographic phenomena which includes global and local autocorrelation. Autocorrelation mainly refers to values of an attribute at close geographical sites which are more similar (positive autocorrelation) or more dissimilar (negative autocorrelation) than values at two distant sites [24,25]. To examine whether spatial patterns are clustered, dispersed, or random, we measured the spatial autocorrelation of environmental complaints based on Global Moran’s I statistic using GeoDa software (version 1.14.0 24, August 2019) [26]. The values of Moran’s I ranges from −1 to 1, where positive values indicate a spatially clustered pattern, negative values imply a spatially dispersed pattern, and zero suggests the random distribution of an attribute, such that there is no spatial autocorrelation. In addition, to identify statistically significant hot spots and clod spots, we further mapped spatial clusters of environmental complaints through calculating the Getis-Ord Gi * statistic with the application of ArcGIS 10.5 software [23,27]. Here, a ‘Queen’ spatial weight matrix was created with 1 order of contiguity for the two spatial statistics so as to identify neighboring prefecture-level city units if their edges or corners were contiguous.

#### 2.2.2. Spatial Regression Models 

Spatial regression models are necessary to consider in explaining the relationship between dependent variables and their potential factors when the observed values of dependent or independent variables present spatial autocorrelation characteristics [28,29]. Spatial regression models have been used in the fields of social economy [30], ecology and the environment [31,32]. Spatial regression models applied in this study include the spatial lag model (SLM) and the spatial error model (SEM).

The spatial lag model takes into account the spatial autocorrelation of the dependent variable, and the SLM is expressed as follows:

(1) $$y={\rho W}_{y}+X\beta +\varepsilon$$

where y is the dependent variables, i.e., the number of cases of environmental complaints for each prefecture-level city; ρ is the spatial autoregressive coefficient; $W_{y}$ is the spatial weight matrix of spatially lagged independent variables, y; X is the n by k matrix of independent variables, wherein n is the number of prefecture-level cities, and k is the number of independent variables; β is the vector of parameter estimates associated with the independent variables; and ε is a vector of random error terms.

The spatial error lag model considers the spatial autocorrelation of random errors. The equations of the SEM is given as:

(2) y=Xβ+ε

(3) $$\varepsilon ={\lambda W}_{\varepsilon }+\mu$$

where λ is the coefficient of spatially lagged random error; $W_{\varepsilon }$ is the spatial weight matrix of spatially lagged error terms, ε; and μ is the random error term with a Gaussian distribution.

The ordinary least square model (OLS), spatial lag model (SLM) and spatial error model (SEM) were all executed using GeoDa software. To determine which model was more suitable to fit the curve—OLS, SLM or SEM—we followed the following criteria of model evaluation [33]: the OLS model was firstly conducted to get the test of Lagrange multiplier (LM) diagnostics for spatial dependence; if the LM-lag was statistically significant whilst LM-error was statistically insignificant, SLM would be the appropriate model. On the contrary, if the opposite scenario occurred, SEM would be chosen. If both LM-lag and LM-error were statistically significant, the model whose Robust LM was more statistically significant should be selected, or else, the results of the OLS model should be kept. Furthermore, the log-likelihood (log-L), R^2^, Akaike information criterion (AIC), and Schwartz criterion (SC) are also used as criterion for selecting the preferred model. A regression model with lower values of AIC and SC, as well as higher values of Log-L and R^2^, would be more likely to be chosen as the most well-fitted model.

## 3. Results and Analysis

### 3.1. Results and Analysis

A total number of 5796 cases of environmental complaints regarding pollution and ecological issues were lodged by citizens directly to the MEP of China via dialing hotline or reporting on the internet for the period of 2013–2017. Among these, 4398 cases were classified as verified complaints, which accounted for 75.88% of the total. That is to say, approximately three quarters of cases were deemed valid complaints indicating regulatory violations of polluting enterprises after the complaint-initiated inspections implemented by local regulators. It also could be seen that citizen environmental complaints were undoubtedly noisy, with an error rate of a quarter (i.e., the proportion of nonverified cases).

**Figure 1:**
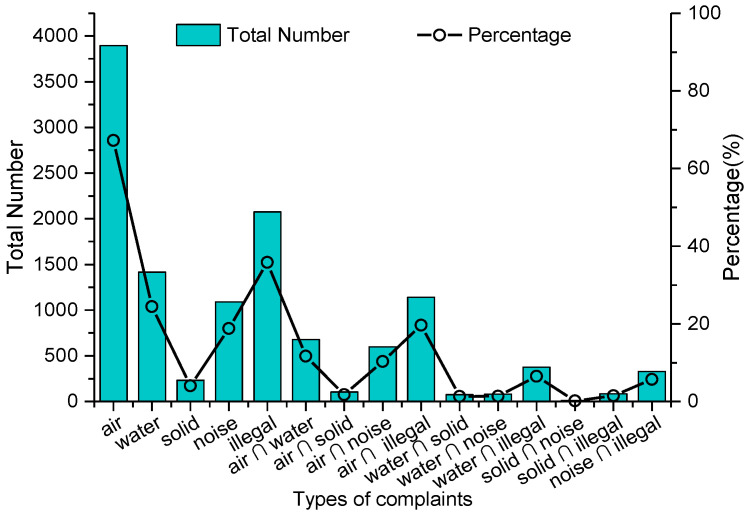
Quantity of different types of environmental problems.

Different types of environmental problems (or pollution) might vary widely in terms of quantity and proportion, while one case could also involve multiple types of environmental issues (Figure 1). Air pollution received the most attention with 3896 cases, which accounted for 67.22% of the total. The proportions of cases associated with water contamination, solid waste pollution, noise pollution, and illegal production were 24.4% (1416 cases), 4.0% (233 cases), 18.8 % (1089 cases), and 35.8% (2079 cases), respectively. In addition, other types, such as emergent environmental incidents, represented only 26 cases, which were excluded from further analysis on pollution types. Figure 1 also presents the intersection characteristics of two types of pollution problems that one case had simultaneously. Along with the process of illegal production, two or more types of pollution issues for one case related to air (1141 cases), water (278 cases), solid waste (85 cases) and noise (330 cases) pollution were filed by local inhabitants. The intersection of air pollution with illegal production, water contamination, and noise pollution ranked in the top three of all two-type cases. Given the accuracy of cases, citizens tended to effectively lodge complaints on certain types of pollution problems. The proportion of verified cases on air pollution (73.23%) and solid waste pollution (73.39%) were closest to the average accuracy; the accuracy rate of 84.11% and 98.36% for types of noise pollution and illegal production, respectively, were both above the average, while water contamination was far below average with a 56.21% accuracy rate. It should be noted that citizens might also complain about an identical enterprise, company or factory that damaged their dwelling environment more than once.

**Figure 2:**
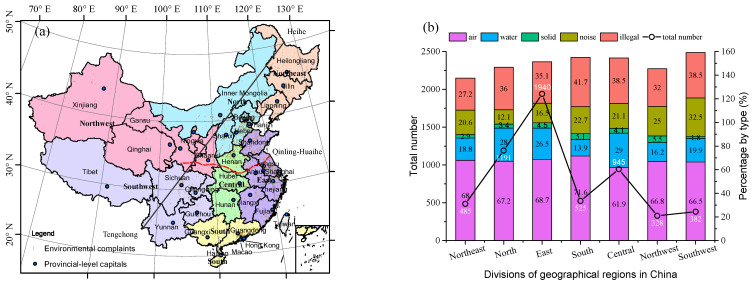
Spatial distribution of citizen environmental complaints. (**a**) Spatial division of China’s geographic regions and spatial visualization of all geocoding cases. (**b**) Spatial distribution of different types of environmental problems for each geographic region in China.

To examine the spatial distribution of 5796 cases of environmental complaints, all provincial-level administrative units in China (including 22 provinces, 5 autonomous regions and 4 municipalities) were divided into 7 geographic regions in light of spatial differentiation of physical and human characteristics (Figure 2a). Moreover, we drew the Heihe-Tengchong Line (also named the Hu Line), which was firstly proposed by Hu Huanyong in 1935 for the interpretation of China’s demographic distribution, and the Qinling-Huaihe Line, which roughly divides China into the two parts of north and south China. The spatial visualization map illustrated the unbalanced distribution of complaint cases, showing that most of them occurred in the densely populated area east of the Hu Line, while a few cases appeared in the sparsely populated areas west of the Hu Line. In particular, plenty of cases were concentrated on three major national urban agglomerations (i.e., Beijing-Tianjin-Hebei Urban Agglomeration, Yangtze River Delta Urban Agglomerations, and Guangdong-Hong Kong-Macao Greater Bay Area from north to south) together with provincial capitals and regional central cities.

Figure 2b showed that, in terms of quantity, the east, north, and central regions of China were the top 3 geographic regions; there were 1940, 1191, and 945 cases in these areas, respectively, which accounted for 70.32% of the total. The rest of the geographic regions, however, only accounted for one third of all cases, with nearly two thirds of national territorial area. East China, the most economically developed coastal regions with a substantial scale of populations and industries, shared the largest portion of the total number of cases in the 7 geographic regions in China. According to statistics at the provincial level, the Ministry of Environmental Protection of China had received numerous cases of environmental complaints from all provinces, except for the Tibet autonomous region. Henan, Hebei, Shandong, Jiangsu, and Guangdong ranked in the top 5 of all provinces and municipalities, contributing to 44.06% of total cases. Unexpectedly, Henan, a central province in China, rather than those more developed eastern coastal regions, occupied the largest proportion of the total number of complaint cases at the provincial level. Besides, the types of environmental problems faced by different geographic regions had similarities in relation to the order of proportions of pollution types on the whole, and differences existed in the actual values of quantity and proportion. Generally, with reference to the current stage of socio-economic development in China, the more economically developed areas would be expected to bring about more complaint cases, indicating a long-term tension in the man-land relationship at the aggregate level.

### 3.2. Geographic Patterns of Environmental Complaints

Despite the fact that the spatial distribution map of all cases presented the overall pattern of citizen environmental complaints, it failed to quantify the pattern and compare different types of environmental problems. Therefore, the Global Moran’s I statistic was calculated for different scenarios at the prefecture-level city scale throughout the country. Results are listed in Table 1. The Moran’s I values for the total, verified and nonverified cases were 0.39, 0.34, and 0.37, respectively, indicating the total, verified and nonverified cases all had a significantly positive spatial autocorrelation. In other words, the spatial distribution of high values and/or low values of cases of environmental complaints was more spatially clustered in terms of the null hypothesis of a randomly distributed pattern. Moreover, all types of environmental problems also had a positive spatial autocorrelation, since their z-scores were statistically significant (*p*-value < 0.001) and positive. The intensity of high and/or low value agglomeration, however, might vary related to different pollution types. The values of z-scores from high to low were as follows: illegal production, water contamination, air pollution, solid waste pollution, and noise pollution. The degree of spatial dependency for air pollution, water contamination and illegal production had similar and much stronger features, whilst the remaining types demonstrated similar but relatively weaker traits of spatial correlation. As a consequence, the spatial regression models were necessarily introduced to investigate the potential factors behind citizens lodging environmental complaints in this study.

**Figure 3:**
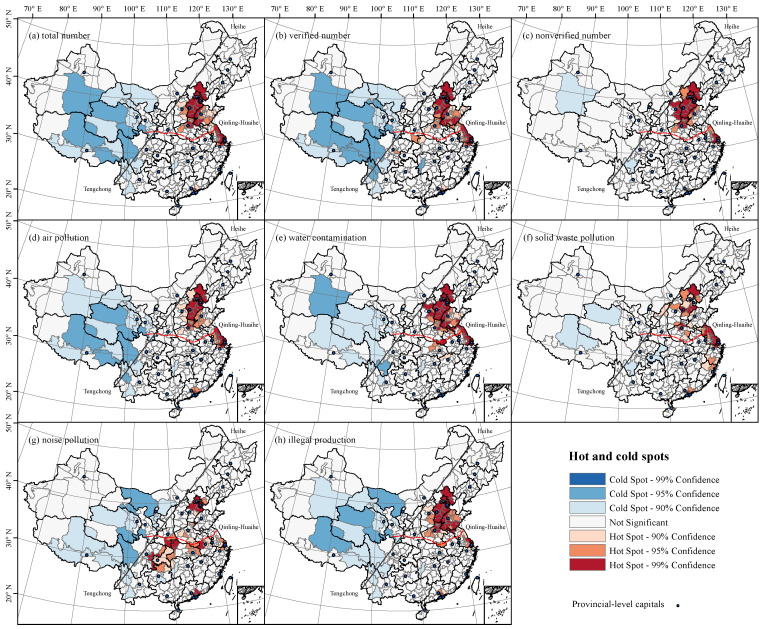
Spatial clusters of hot and cold spots of environmental complaints in China.

The spatial clusters of hot and cold spots of citizen environmental complaints at the prefecture level were mapped and presented in Figure 3. Overall, the hot spots of the total cases were mostly distributed in the area east of the Hu Line, whereas the area west of the Hu Line contained the majority of cold spots. Indeed, the spatial clusters of hot spots could be roughly divided into three major parts in terms of the discrepancies of location and scale, which was spatially consistent with the three major national urban agglomerations in China. The largest cluster of hot spots was observed in the North China Plain, spreading over the Beijing-Tianjin-Hebei agglomeration, along with Shanxi, Shandong and Northern Henan. The second largest cluster was the core parts of the Yangtze River Delta Urban agglomerations, mainly including Shanghai, Jiangsu, and northern Zhejiang. The Pearl River Delta cluster consisted of main cities like Guangdong, Dongguan, and Shenzhen that featured a highly concentrated but small range.

In addition, the verified cases shared a similar spatial pattern with the total, indicating that the spatial pattern depicted by the total cases with noisy information could also be used in the identification of pollution areas or problems. Furthermore, the hot spot clusters of the nonverified cases were slightly different from the total and verified cases, suggesting that the greater the number of cases of complaints, the more noisy information would be generated, and thus more inspections and governance resources would be allocated to these hot spot areas. However, these noisy cases were not ‘noisy’ anymore. In fact, they could also be a unique indicator in reflecting the status of regional environmental quality, because noisy cases might reflect citizens’ concerns and demands on the environment to some extent. With respect to different types of pollution issues, the hot spots of air pollution, water contamination, solid waste pollution, and illegal production had a similar spatial pattern on the whole, while the hot spots of noisy pollution exhibited a more discrete spatial pattern that was mainly located in the south area of the Qinling-Huaihe Line. 

It is worth noting that the spatial patterns delineated by environmental complaints may be biased in reflecting real regional pollution and citizen concerns regarding the environment. This is mainly because citizen environmental complaints can be considered a product of various factors involving socio-economic levels, environmental pollution discharges and citizen environmental awareness.

### 3.3. Results of Regression Models

In this study, the number of cases of environmental complaints for prefecture-level cities were selected as the dependent variables. The dependent variables were then classified as three scenarios (the total, the verified, and the nonverified cases) and five types of environmental problems. After a thorough review on the literature of citizen environmental complaints, we selected 10 independent variables from three aspects to test the relationship between environmental complaints and its potential factors. Population, GDP, and industrial structure of secondary and tertiary industries were defined as four proxy variables to represent socio-economic status. Three pollutants—industrial waste water, sulphur dioxide, and industrial soot (dust)—were selected as the proxy variables of environmental pollution. With respect to environmental awareness, three proxy variables regarding the telephone, internet and education were chosen. It should be noted that the independent variables were summarized from numerous spatial points of 286 prefecture-level cities so as to match the spatial scale of the influencing factors. Descriptive statistics for independent and dependent variables were summarized and listed in Table 2. 

To test the multicollinearity problem of the independent variables above, we performed an analysis of the variance inflation factor (VIF) before model fitting using the IBM SPSS Statistics 26 software. The results of the VIF analysis of all explanatory variables were all less than 10, indicating no obvious multicollinearity existed in our regression models. Hence, these 10 proxy variables could be employed in the subsequent model regression and analysis. 

Model selection is the critical process of determining which model will be chosen as the appropriate one for estimation. The tests of Lagrange multipliers (LM) and Robust LM for spatial dependence were conducted; Table 3 provides the test results of three scenarios for the total, the verified, and the nonverified cases. For the total cases, the test statistics of both LM-lag and LM-error were highly significant, whereas Robust LM-error (*p*-value < 0.0001) was more significant than that of Robust LM-lag (*p*-value = 0.0003). Meanwhile, the R^2^ and Log-L of the SEM were both higher than that of the SEM, whilst the AIC and SC were lower than that of the SEM (Table 4). In addition, the spatial autoregressive coefficient ρ and the coefficient of random error λ were statistically significant, indicating that spatial regression models were necessary to be adopted to replace the traditional nonspatial model, the OLS (Table 4). Therefore, according to the criteria of model selection above, SEM was selected as the appropriate model for the total cases to explore the effects of influencing factors on citizen environmental complaints. Likewise, we also selected the most fitted model for the remaining scenarios and all types of environmental problems on the basis of the same model selection criteria.

As shown in Table 4, we provided the results of the OLS, SLM, and SEM for the total cases, whereas the appropriate model results were chosen and presented only for the rest of the verified and nonverified scenarios, as well as for all the five types involved.

The results of the OLS, SLM and SEM show that the explanatory power for the variations in the total cases of citizen environmental complaints were 64%, 72% and 74%, respectively (Table 4). This suggested that the explanatory power of the spatial regression models (SLM and SEM) would be much stronger than that of the traditional nonspatial model (OLS) after considering the spatial lag or spatial error terms. The values of ρ (0.40) and λ (0.58) parameters were both higher and statistically significant (Table 4); the former indicated a positive spatial feedback effect, i.e., a greater number of cases in a city might raise the predicted cases of its neighboring cities. The latter showed that the unexplained variation in citizen complaints was positively correlated, and this could be attributed to the results of omitted variables that affected cases lodged by citizens between adjacent cities. Apparently, the latter tended to dominate the best position to better understand how these potential factors exerted influences on the occurrence of citizen complaints in China.

From the perspective of socio-economic status, only per capita GDP was significantly and negatively associated with the total number of cases of citizen environmental complaints. This indicated that the total number of citizen complaints decreased as per capita GDP increased. It also implied that, to a large extent, economic levels were not spatially consistent with environmental pollution at prefecture levels. In terms of the spatial clusters above, a relatively small number of hot spots were distributed in economically developed coastal cities of eastern China or located in the cities of the southern China, which have a high environmental and ecology carrying capacity. Generally, this was inconsistent with several studies performed using provincial-level datasets for the past 10 years or longer. Their results show that per capita GDP or household income level was positively associated with complaints [10,19,20]. Furthermore, it could also be seen that the coefficients of population density, together with the proportion of secondary and tertiary industry in GDP, were all statistically insignificant.

With regard to the aspect of environmental pollution, the positive correlation coefficient of the volume of industrial wastewater and SO_2_ with the total cases suggested that exposure to these harmful pollutants increased the likelihood of citizen complaints. An increase of 1% in industrial wastewater discharge (10,000 tons) and SO_2_ (1 ton) seemed to induce an increase of approximately 5.5 cases and 0.5 cases, respectively. Industrial soot (dust) emission was positive but not significant, indicating that even pollutants with highly visible particulates were not necessarily significantly related to the number of environmental complaints. This confirmed the results of previous studies, most of which concluded that the volume or intensity volume of certain pollutant discharges in a region determined the number or incidence of citizens lodging environmental complaints [10,15,19,20]. Therefore, the effects of different pollutants on citizen behaviors regarding filing complaints, or, in other words, how regional environmental pollution defined the pattern and processes that triggered individuals or communities to make complaints, seemed to become more complex than expected. 

For environmental awareness, two variables of communication tools were found to exert impacts on the probability of environmental complaints. The coefficient of the mobile telephone was negative, and the coefficient was positive for the internet. This implied that a greater number of mobile telephones decreased the number of the total cases; conversely, the more accessible the internet, the more cases would be generated. The proliferation of phones and the internet not only enriched the methods of complaints, but also raised citizen awareness regarding environmental protection. Moreover, the coefficient of educational levels of citizens was positive but insignificant. There was no firm evidence to show that citizens with higher education background might care more about pollution discharges or have a stronger willingness to pay for the practice of environmental protection at the prefecture level. Unexpectedly, this finding did not share the same view with previous studies, which believed people with higher educational levels would have a stronger propensity to complain [15,18,20].

As for the verified cases of citizen complaints, the coefficients of all independent variables were similar to that of the total cases in light of significant levels and direction of effects. Hence, the total cases, with or without verification, could be regarded as the dependent variable to investigate the effects of potential factors behind the citizen behaviors lodging complaints. The comparative analysis, to some extent, could be regarded as the validation process in data validity and results reliability. The reason might be ascribed to the high proportion of verified cases in the total. However, for the nonverified cases, only two variables of communication tools were statistically significant at the 1% significant level with the opposite effects. Unlike traditional methods of citizen complaints, lodging complaints online via PC or mobile apps was more time saving and cost effective for local inhabitants to reflect their concerns and demands regarding environmental issues. Accordingly, an increasing amount of nonverified complaint cases (or inaccurate cases) would be inevitably lodged by citizens to the authorities due to the shifting transition of complaint channels.

In addition, different pollutants discharged into ambient media might result in corresponding pollution types [10]. For example, industrial waste gases emitted into the air were likely to bring about air pollution, whereas industrial wastewater or domestic sewage dumped into the rivers, lakes, or seas would cause water pollution. Nevertheless, this causality might not live up to our expectation sometimes. To test the casual relationship in citizen complaints, therefore, we conducted the same model fitting for each type of pollution problems and presented the most suitable model only (Table 4). The results showed that the volume of industrial waste discharged and sulphur dioxide emissions had significant impacts on the number of the total cases of air pollution. This result indicated that a type of problem might be induced by multiple pollutants at the regional or macro level. Furthermore, the volume of industrial wastewater discharged was significantly associated with the total cases of air pollution, solid waste pollution, and illegal production, in addition to water contamination. This finding suggested that certain pollutants might lead to various types of problems filed by local residents, but not necessarily contributed to its corresponding polluting issues. Similar to the three scenarios above, the number of subscribers to internet services also had significantly positive effects on the likelihood of citizen complaints for all types. It is worth nothing that the results of all types might be affected by the sample size of cases, which possibly caused instability in estimation.

## 4. Discussion

**Figure 4:**
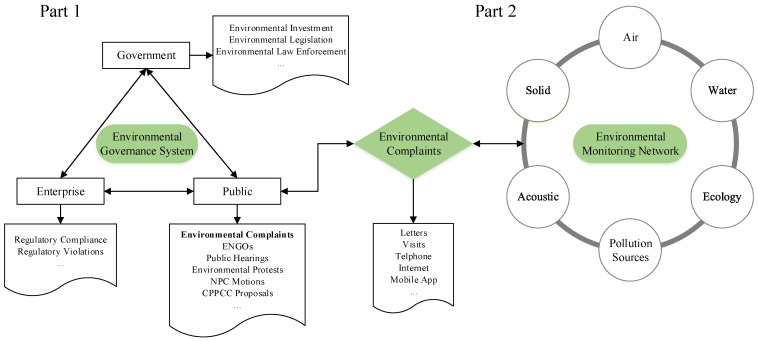
An analysis framework of the role of citizen environmental complaints in China’s environmental governance system and environmental monitoring network.

Citizen environmental complaints play a key role in China’s environmental governance system and environmental monitoring network. In recent years, the status of ecology and the environment in China has been gradually improved as a whole, and at the same time the citizens’ concerns and demands for environmental quality has also been continually rising [20]. Moreover, rapid changes in technology, such as mobile internet, artificial intelligence (AI), and big data, facilitated citizens filing complaints and regulators collecting information. Hence, the number of cases of citizen complaints seems to be bound to continue to expand in the near future, which may lead to plenty of resources allocated to responding to and solving these issues coming from citizens over the country. In this context, it is extremely necessary to reevaluate the role of citizen environmental complaints in China. To vividly depict its role in the above two systems, we illustrated an analysis framework of the role of environmental complaints lodged by citizens via internet and hotlines in China (Figure 4).

As mentioned above, despite the fact that the effectiveness of citizen complaints on environmental performance remains contentious, it is certain that it can be considered as an essential and indispensable part of China’s environmental governance system. As shown in Part 1 of Figure 4, the subsystem of public participation with multiple bottom-up channels radically changes the situation wherein the government acts as the sole agent for environmental health and safety, as in the governance system illustrated by a top-down hierarchical architecture [10]. Compared with other channels, citizen environmental complaints today are typically characterized by convenient, cost-effective, real-time, relatively moderate, complaint-driven pollution abatement, and the real public participation involved the majority of people. The system works in two ways, directly or indirectly, compelling polluters to adhere to laws, regulations and standards. On the one hand, citizens, individuals, or communities in China, once damaged by pollution, can lodge their complaints to the authorities; after receiving cases of complaints, the agencies and regulators are very responsive to citizens’ concerns on environmental issues by inspecting the potential polluters within a given time. If the complaint is found to be true, agencies and regulators will deal with the regulation violations of polluting enterprises in accordance with the law and regulations by, for example, demanding rectification within a specific time, suspending production, imposing fines or penalties, banning or closing the enterprise, and the like. It has also been proven that environmental complaints could significantly promote the law enforcement of the government only [6]. On the other hand, the public may also enhance their bargaining position by threatening to make complaints to the authorities when they are directly engaged in negotiation with neighboring polluting enterprises [10]. An error rate of approximately a quarter in citizen complaints may result in a huge waste of inspection resources, but this seemingly ‘unnecessary’ practice is actually inevitable, and extremely needed to promote environmental justice and environmental democracy in China in terms of resolving individual problems regarding environmental pollution.

Over the past few decades, China’s own environmental monitoring network has been built up by the government, which mainly refers to monitoring pollution sources, ecology, coastal areas, and the atmospheric, water, soil, and acoustic environment, as described in Part 2 (Figure 4). This network is comprised of many fixed monitors. Currently, these include 2100 air quality monitoring stations, 2767 surface water monitoring sections, 300 water quality automatic stations, and more than 40,000 soil monitoring points [34]. Citizen environmental complaints, as a special monitoring tool, are also characterized by large coverage, high spatial-temporal resolution, noisy information. This sort of ‘noisy’ feature possibly constraints its effective information provision as environmental indicators that can be further interpreted from several aspects, as follows. Firstly, unlike those precise fixed monitors that can detect subtle changes in the environment and provide a variety of quantitative indicators, citizen complaints are born to be subjective and qualitative when regarded as a monitoring tool. Meanwhile, the complexity and diversity of environmental pollution reinforces its uncertainty in reflecting the true status of the environment and ecology. Secondly, people often make judgments on environmental issues basically by relying on personal senses (i.e., sight, smell, touch, taste, and hearing) and, therefore, visible, odorous, and loud pollutants or pollution processes tend to receive more attention and even be exaggerated by personal perception to some extent. Nevertheless, it is scarcely possible for citizens without the relevant expertise to recognize those invisible, odorless, or imperceptible pollutants that may truly damage the ecology and environment; this includes, for instance, ecological degradation, soil heavy metal pollution, and agricultural non-point source pollution. Finally, individual or regional divergence related to economic, education, pollution, and governance capabilities will project this noisy information on the map, and this biased signal of citizen complaints, to some extent, may be further strengthened or weakened at different scales. In practice, the approximately 75% accurate rate of China’s citizen complaints on environmental issues was much higher than that of the 55% rate of verified cases in the study of citizen complaints and livestock operations in Michigan, USA [17].

Socio-economic characteristics are assumed to be the basis to influence and, to a large extent, determine overall pollution status, citizen awareness or perception, and even governance capability. In a previous study, the EKC effect was found between income levels and the number of environmental complaints, with the turning point of around 140,000 yuan, which China was far from reaching [20]. In our conclusions, however, the coefficient between per capital GDP and the number of citizen complaints was statistically negative. However, this did not necessarily indicate that an inverted U-shaped curve had been in existence in our study. In the past decade, China’s economic model, which had been undergoing high-speed development and was energy-intensive and highly-polluting, was already being progressively transformed into high-quality development with lower carbon emissions and pollution discharges [35,36]. Furthermore, accompanying industrial transformation and upgrades in eastern coastal areas in China, polluting industries and sectors had already begun to transfer to central and western interior regions, which urgently need to develop their economy and have loose environmental policies and regulations [37,38]. Additionally, the map of regional economies displayed the rising disparity between the northern and southern areas in China owing to regional differences in economic development models [39,40]. In contrast to the northern areas, southern China went through a more thorough reform, opening up, and marketization. Given that regional differentiation, the environmental capacity, resources endowment and ecology in the southern and coastal regions is apparently much better than that of the rest of China. Therefore, a negative effect was found between per capita GDP and citizen complaints at the prefecture level.

Theoretically, no pollution should result in no complaints. Nearly all studies had firmly confirmed that environmental pollution triggered citizens lodging complaints. In terms of pollution types, air quality or pollution received the most attention in China, which has been proven by other studies [10,15]. China has been facing the worst air pollution problem in the world [41,42]. Air quality changes can be easily sensed by citizens anytime and anywhere [10,15]. In addition, other types of pollution, for example, water pollution, might also deteriorate air quality by emitting odorous gases [15,21]. Nevertheless, it has been rather difficult for an agreement to be reached on the effects of specific pollutants on complaints based on various data sources and methods. Unexpectedly, there was no significant relationship between the volume of industrial soot (dust) emission and the number of complaints, even if in terms of air pollution. This result was contradictory with the study conducted by Dasgupta and Wheeler [10], which suggested that high-visible dust (particulate) intensity could consistently and significantly induce citizens filing complaints. Actually, environmental pollution, or a type of pollution, is generally represented by a set of indicators; air pollution, for example, can be systemically measured by various indicators of SO_2_, NO_2_, CO, O_3_, PM10, PM 2.5, etc. Furthermore, China, especially in northern areas, has been always covered by large-scale fog and haze with poor visibility and toxic substances in winter, which root in a large number of production and living activities, such as coal-fired heating, motor vehicles and traditional heavy industry enterprises [43,44]. Given that, hardly positioning these pollution sources and ‘free riding’ among citizens might also result in an insignificant association relationship. It should be noted that there was still a gap between individual cases and regional environment quality due to the existence of the ecology fallacy of scale transformation.

The era of the internet has brought unprecedented changes to all walks of life in China, and environmental governance of course is not an exception. On the one hand, the internet provides advanced media technology of information disclosure and access for the government and the public, as well as multiple platforms for citizens expressing their concerns and demands. In late 2011, the US Embassy reported Beijing’s PM 2.5 monitoring data on Twitter, when PM 2.5 was not yet a regulated indicator or pollutant in China at the time [20]. The PM 2.5 monitoring dynamics were reposted on Sina Weibo (a social media website in China) by a netizen, which aroused a furious debate over air quality among scholars, officials, and netizens throughout the country. In practice, this event could be regarded as a landmark for the rising environmental awareness of Chinese citizens. As a consequence, the debate derived on the internet directly prompted the government to set up a national network for PM2.5 monitoring, and the corresponding laws, regulations and other policies were enacted and implemented in order to alleviate citizen grievances and improve air quality. On the other hand, the government also established the 12369 platform based on internet and mobile apps in 2011, which greatly facilitated citizens lodging complaints. With a few clicks of buttons and the typing of a few lines of text, citizens in China can accomplish the whole procedure of an environmental complaint case. In fact, the proliferation of the internet and telephone, to a great extent, lower the threshold of the educational level that early complaint channels of visits or letters should have. This could be an appropriate reason to explain why the effect of higher education on citizen complaints was insignificant in this study.

The government of China should continue to encourage and expand public participation with regard to environmental issues and raise the status of citizen complaints in environmental governance. Citizen environmental complaints should work together with other policy instruments, such as command-control policies, market incentive policies and other modes of public participation. For example, the role of citizen complaints can be viewed as an important reference for decision making in the environmental governance of the government in terms of investment, legislation and enforcement. In order to improve environmental performance, the central government sets a series of binding and anticipatory targets or goals that are further diluted to subordinate administrative units to perform the tasks from top to bottom. In the process of responding to citizen complaints, local agencies and regulators ought to combine complaint-initiated inspections on polluters with the attainment of these ambitious targets, such as energy consumption, carbon emissions, and air and water quality. One thing that must be kept in mind is the information privacy of complainants, which urgently need to be further protected and managed by the authorities. It has been reported that, in early 2021, Mr. Shao, who lived in Zhoukou city, Henan province, dialed a hotline to complain about midnight pollution emissions of a factory to the local environmental protection agency; nevertheless, he was assaulted, beaten and threatened by the employees of this polluting factory owing to the leakage of personal information by the local regulators [45].

In reality, tens of thousands of citizens can be seen as innumerable sensors monitoring environmental dynamics; therefore, China has the largest environmental monitoring network based on individuals in the world. The synergy and coordination between citizen environmental complaints and other monitoring instruments need to be promoted in years to come. For example, air quality monitoring stations often fail to identify the position of various polluters, while local residents are familiar with their neighboring status and can easily determine the pollution sources or problem areas, especially for those small, dispersed polluters and for remote, rural areas in China. Hence, their functions can complement each other in providing more reliable and better information on the environment and ecology. In order to reduce noisy information, it is necessary to introduce advanced technology and deeply combine this with China’s current major social media platforms, such as Wechat, Sina Weibo, and Douyin (TikTok). It will be helpful for regulators to scientifically interpret environmental issues through providing richer information regarding text, pictures, short videos, or even live broadcasts. In addition, the cultivation of environmental awareness should be strengthened, especially for those relatively poorer communities and remote areas, by means of increasing household income, enhancing basic education, building internet infrastructure, etc.

## 5. Conclusions

Based on 5796 cases of environmental complaints lodged by citizens via hotlines and the internet to the MEP of China, we examined the spatial characteristics and influencing factors of complaints for the period 2013–2017. The roles of citizen complaints in two systems were then reevaluated. Among all cases, 75.88% of cases were identified as verified complaints, while nearly a 25% noisy rate directed large amounts of inspection resources to be utilized in response to nonverified cases. Different types of citizen complaints varied widely in terms of quantity and proportion; air pollution received the most attention, accounting for 67.22% of the total. The hot spots of citizen complaints were mostly distributed in the three major national urban agglomerations in China, and the clusters from large to small scale were the Beijing-Tianjin-Hebei, the Yangtze River Delta, and the Pearl River Delta urban agglomerations, respectively.

Spatial econometric models could improve the estimation effects between citizen complaints and their potential factors. We found that industrial wastewater and SO_2_ were positively associated with the likelihood of citizens lodging complaints, while the effect of industrial soot (dust) emission was insignificant. Citizen complaints might be triggered by certain, but not all, forms of pollutants, even though highly visible particulate pollutants did not necessarily induce corresponding complaints. Moreover, the negative relationship between citizen complaints and per capita GDP revealed the unbalanced geographical pattern between economic development and environmental quality. In addition, the proliferation of the internet greatly facilitated citizens filing complaints through improving information access, simplifying complaint process, and even lowering the threshold of educational level for lodging complaints. Citizens’ environmental complaints should be deeply combined with major social media platforms (e.g., TikTok, Twitter, Facebook) in the world so as to provide richer and more diverse data. The potential applications should be investigated; for example, it can be applied in the synergy of pollution and carbon emission reductions as a kind of monitoring or policy tool in climate change research. Additionally, it will be helpful to observe and assess the process of achieving Sustainable Development Goals (SDGs) regarding environmental issues, especially for those developing countries in the world. Finally, the synergy mechanism between citizen environmental complaints and other parts of China’s environmental monitoring network and environmental governance system should be established in the near future.

## Figures and Tables

**Table 1:** The Global Moran Index of environmental complaints in China.

	Total	Verified	Nonverified	Air	Water	Solid	Noise	Illegal
Moran’s Index	0.39	0.34	0.37	0.36	0.40	0.19	0.16	0.40
Expected Index	−2.90 × 10^−3^	−2.90 × 10^−3^	−2.90 × 10^−3^	−2.90 × 10^−3^	−2.90 × 10^−3^	−2.90 × 10^−3^	−2.90 × 10^−3^	−2.90 × 10^−3^
Mean	−3.10 × 10^−3^	−3.20 × 10^−3^	−2.80 × 10^−3^	−3.10 × 10^−3^	−2.30 × 10^−3^	−2.30 × 10^−3^	−3.30 × 10^−3^	−3.30 × 10^−3^
SD	3.27 × 10^−2^	3.33 × 10^−2^	3.27 × 10^−2^	3.24 × 10^−2^	3.58 × 10^−2^	3.44 × 10^−2^	3.27 × 10^−2^	3.37 × 10^−2^
Z-score	11.88 ***	10.44 ***	11.26 ***	11.26 ***	11.35 ***	5.55 ***	5.03 ***	11.82 ***

***, *p* < 0.01.

**Table 2:** Descriptive statistics of selected variables for 286 prefecture-level cities.

Variable	Min.	Max.	Mean	Std. Dev	Description
the total	0.00	254.00	19.59	22.97	the total number of complaint cases
the verified	0.00	159.00	14.80	16.19	the verified number of complaint cases
the nonverified	0.00	95.00	4.79	8.07	the nonverified number of complaint cases
air	0.00	172.00	13.13	15.64	the total number of complaint cases of air pollution
water	0.00	59.00	4.84	6.68	the total number of complaint cases of water contamination
solid	0.00	8.00	0.77	1.22	the total number of complaint cases of solid waste pollution
noise	0.00	36.00	3.67	4.46	the total number of complaint cases of noise pollution
illegal	0.00	87.00	7.08	9.03	the total number of complaint cases of illegal production
POPD	5.77	2501.14	436.30	337.85	population density (person/sq.km)
PerGDP	1.10	20.72	5.11	2.96	per capita GDP (10,000 yuan)
SIP	15.17	71.45	46.67	9.57	secondary industry as percentage to GRP (%)
TIP	24.17	79.65	40.99	8.70	tertiary industry as percentage to GRP (%)
Water	0.01	6.05	0.66	0.72	volume of industrial waste water discharged (10,000 tons)
SO_2_	0.02	42.68	4.93	4.25	volume of sulphur dioxide emission (tons)
Soot	0.09	185.99	4.88	13.91	volume of industrial soot (dust) emission (tons)
Tel	43.00	4052.00	457.28	498.36	number of subscribers of mobile telephones at year-end (10,000 households)
Internet	6.00	1205.00	89.08	118.18	number of subscribers of internet services (10,000 households)
Student	4.79	1293.69	192.87	254.39	students enrollment of regular institutions of higher education per 10,000 persons

**Table 3:** Diagnostics for spatial dependence.

TEST	The Total		The Verified		The Nonverified	
	MI/DF	VALUE	MI/DF	VALUE	MI/DF	VALUE
Moran’s I (error)	0.36	9.54 ***	0.28	7.67 ***	0.31	8.43 ***
Lagrange Multiplier (lag)	1.00	69.65 ***	1.00	39.52 ***	1.00	72.00 ***
Robust LM (lag)	1.00	8.63 ***	1.00	4.07 **	1.00	11.62 ***
Lagrange Multiplier (error)	1.00	81.69 ***	1.00	52.03 ***	1.00	63.26 ***
Robust LM (error)	1.00	20.67 ***	1.00	16.58 ***	1.00	2.88 *
Lagrange Multiplier (SARMA)	2.00	90.32 ***	2.00	56.10 ***	2.00	74.88 ***

*, *p* < 0.1; **, *p* < 0.05; ***, *p* < 0.01.

**Table 4:** Regression model results.

Variable	The Total			The Verified	The Nonverified	Air	Water	Solid	Noise	Illegal
	OLS	SLM	SEM	SEM	SLM	SEM	SEM	SEM	OLS	SEM
CONSTANT	3.93	7.76	16.34	7.23	3.17	11.15	8.70 *	0.44	4.54 *	2.71
POPD	6.22 × 10^−3^ *	8.00 × 10^−4^	−3.06 × 10^−5^	1.73 × 10^−4^	2.48 × 10^−4^	4.28 × 10^−4^	−1.08 × 10^−4^	3.22 × 10^−4^	9.49 × 10^−4^	2.12 × 10^−3^
PerGDP	−0.63	−0.74 **	−0.91 **	−0.73 ***	−0.13	−0.44	−0.26 *	−0.02	−0.29 ***	−0.53 ***
SIP	0.07	−0.03	0.04	0.03	4.54 × 10^−2^	0.01	−0.02	0.01	−0.01	0.05
TIP	−0.10	−0.20	−0.27	−0.10	−0.09	−0.20	−0.12	−0.01	−0.09 **	−0.05
Water	4.54 ***	4.21 ***	5.42 ***	4.77 ***	0.34	3.04 ***	1.19 *	0.51 ***	0.60	1.96 ***
SO_2_	0.72 ***	0.66 ***	0.45 **	0.50 ***	0.06	0.38 **	0.07	−0.02	0.22 ***	0.23 **
Soot	3.64 × 10^−2^	−4.38 × 10^−4^	−1.72 × 10^−2^	8.05 × 10^−3^	−1.23 × 10^−2^	−2.26 × 10^−2^	4.08 × 10^−3^	4.50 × 10^−3^	−6.07 × 10^−3^	4.97 × 10^−3^
Tel	−6.10 × 10^−3^ *	−8.53 × 10^−3^ ***	−5.71 × 10^−3^ **	−1.01 × 10^−3^	−5.96 × 10^−3^ ***	−2.63 × 10^−3^	−1.59 × 10^−3^	−2.04 × 10^−4^	2.32 × 10^−3^ ***	−1.44 × 10^−3^
Internet	0.15 ***	0.15 ***	0.14 ***	0.09 ***	0.06 ***	0.10 ***	0.03 ***	2.24 × 10^−3^ **	0.01 ***	0.04 ***
Student	2.59 × 10^−4^	6.05 × 10^−3^	4.79 × 10^−3^	1.70 × 10^−3^	2.42 × 10^−3^	1.68 × 10^−3^	−1.44 × 10^−3^	−3.45 × 10^−4^	4.27 × 10^−3^ ***	4.25 × 10^−4^
ρ	—	0.40 ***	—	—	0.45 ***	—	—	—	—	—
λ	—	—	0.58 ***	0.51 ***	—	0.55 ***	0.54 ***	0.28 ***	—	0.50 ***
R^2^	0.64	0.72	0.74	0.74	0.57	0.74	0.50	0.22	0.53	0.61
Log-L	−1156.37	−1125.59	−1120.54	−1021.35	−890.14	−1011.02	−860.51	−430.61	−724.71	−909.08
AIC	2334.75	2275.18	2263.07	2064.70	1804.28	2044.04	1743.02	883.22	1471.42	1840.16
SC	2374.96	2319.05	2303.29	2104.92	1848.15	2084.26	1783.23	923.43	1511.64	1880.37

*, *p* < 0.1; **, *p* < 0.05; ***, *p* < 0.01.

## Data Availability

The data presented in this study are available on request from the author. The data are not publicly available due to privacy. Images employed for the study will be available online for readers.
